# Epilepsy Syndromes in the First Year of Life and Usefulness of Genetic Testing for Precision Therapy

**DOI:** 10.3390/genes12071051

**Published:** 2021-07-08

**Authors:** Allan Bayat, Michael Bayat, Guido Rubboli, Rikke S. Møller

**Affiliations:** 1 Department of Regional Health Research, University of Southern Denmark, DK-5230 Odense, Denmark; rimo@filadelfia.dk; 2 Danish Epilepsy Centre, Department of Epilepsy Genetics and Personalized Medicine, DK-4293 Dianalund, Denmark; guru@filadelfia.dk; 3 Department of Clinical Genetics, University Hospital of Aarhus, DK-8200 Aarhus, Denmark; bayat86@hotmail.com; 4 Department of Neurology, University Hospital of Aarhus, DK-8200 Aarhus, Denmark; 5 Institute of Clinical Medicine, University of Copenhagen, DK-1165 Copenhagen, Denmark

**Keywords:** epilepsy genetics, diagnostic yield, early-onset epilepsy, epileptic encephalopathy, benign and self-limiting (familial) epilepsy syndromes, precision therapy

## Abstract

The high pace of gene discovery has resulted in thrilling advances in the field of epilepsy genetics. Clinical testing with comprehensive gene panels, exomes, or genomes are now increasingly available and have led to a significant higher diagnostic yield in early-onset epilepsies and enabled precision medicine approaches. These have been instrumental in providing insights into the pathophysiology of both early-onset benign and self-limited syndromes and devastating developmental and epileptic encephalopathies (DEEs). Genetic heterogeneity is seen in many epilepsy syndromes such as West syndrome and epilepsy of infancy with migrating focal seizures (EIMFS), indicating that two or more genetic loci produce the same or similar phenotypes. At the same time, some genes such as *SCN2A* can be associated with a wide range of epilepsy syndromes ranging from self-limited familial neonatal epilepsy at the mild end to Ohtahara syndrome, EIFMS, West syndrome, Lennox–Gastaut syndrome, or unclassifiable DEEs at the severe end of the spectrum. The aim of this study was to review the clinical and genetic heterogeneity associated with epilepsy syndromes starting in the first year of life including: Self-limited familial neonatal, neonatal-infantile or infantile epilepsies, genetic epilepsy with febrile seizures plus spectrum, myoclonic epilepsy in infancy, Ohtahara syndrome, early myoclonic encephalopathy, West syndrome, Dravet syndrome, EIMFS, and unclassifiable DEEs. We also elaborate on the advantages and pitfalls of genetic testing in such conditions. Finally, we describe how a genetic diagnosis can potentially enable precision therapy in monogenic epilepsies and emphasize that early genetic testing is a cornerstone for such therapeutic strategies.

## 1. Introduction

Although epilepsy can result from factors such as stroke, asphyxia, infections, autoimmune disorders, trauma, and tumors, a significant number of subjects with epilepsy are thought to have an underlying genetic factor [1]. Recent developments in molecular genetics have improved our understanding of the genetic causes, contributors, and modifiers of epilepsy. Next-generation sequencing has led to an explosion of gene discoveries in human disorders and up to 50% of monogenic epilepsies reach a precision diagnosis [2]. The highest diagnostic yield is usually amongst subjects with early-onset seizures and a global neurodevelopmental delay [3,4]. New techniques that enable investigation of the epigenetic regulation and longer read lengths [2] combined with functional testing of variants of unknown significance, even in known genes, and systematic match-making exchange [5] are needed to facilitate a genetic diagnosis for the remaining half. The incidence of epilepsy is high during the first year and then declines throughout childhood [6]. The clinical pictures and prognoses of early-onset monogenic epilepsy syndromes are diverse and include both benign and self-limited (familial) epilepsy syndromes as well as devastating developmental and epileptic encephalopathies. Genetic testing is essential for determining precision medicine strategies and to prevent unnecessary and potentially harmful diagnostic procedures and managements. A genetic diagnosis can also provide useful information on the natural history and prognosis of a disease and facilitate more targeted genetic counseling. Furthermore, it allows the subject and their family to enter gene-specific networks of families with the same condition.

We review the phenotypic spectrum and genetic landscape of electroclinical epilepsy syndromes that begin during the first year of life (Table 1). We also discuss the utility of different genetic test strategies and provide examples of how genetic testing can enable precision therapy approaches in monogenic disorders.

## 2. Benign and Self-Limited (Familial) Epilepsy Syndromes in Infancy

In these syndromes, de novo and inherited pathogenic variants produce broadly similar electroclinical features in both familial and non-familial cases. Therefore, the inheritance is assigned as a secondary denominator and is placed in parenthesis. Five benign epilepsy syndromes are recognized in infancy: Benign (familial) neonatal epilepsy (BFNE), benign (familial) neonatal-infantile epilepsy (BFNIE), benign (familial) infantile epilepsy (BFIE), genetic epilepsy with febrile seizures plus (GEFS+) spectrum, and myoclonic epilepsy in infancy [7,8,9]. All syndromes typically occur in previously healthy children with age-appropriate development, examination, and head circumference. Development, cognition, and interictal electroencephalograms (EEGs) are normal, and subjects respond well to antiseizure medications [7]. While these syndromes are typically considered to be self-limiting this may not always be the case.

### 2.1. Benign (Familial) Neonatal Epilepsy

BFNE is a condition characterized by recurrent seizures in otherwise healthy newborn babies. The seizures begin around day 5 of life and usually remit by the end of the first year [10]. Subjects display normal neuropsychological development and EEG recordings. BFNE is a genetically heterogeneous disorder due to loss-of-function variants in the *KCNQ2* or *KCNQ3* genes. These two genes encode voltage-gated potassium channel subunits Kv7.2 and Kv7.3.7 [10,11]. Unprovoked focal onset motor seizures that evolve to bilateral tonic-clonic seizures are the predominant seizure type, and they are occasionally associated with non-motor symptoms such as staring, apnea, and cyanosis [7]. Historically, the percentage of children with BFNE who experience subsequent (i.e., post-infantile) focal or generalized seizures has been thought to be 10–18% [10,12,13]. Seizures re-occur in different age groups spanning from early childhood to adulthood, with most events in childhood being induced by fever and most seizures in adolescence and adulthood being afebrile [13].

### 2.2. Benign (Familial) Neonatal-Infantile Epilepsy

BFNIE occurs between the first day and 7 months of life (mean 11 weeks), and symptoms tend to remit by the end of the first year (17). Symptoms resemble BFNE, and the overlap in age at seizure onset makes genetic testing crucial. BFNIE is often caused by a missense variant in *SCN2A* encoding voltage-gated sodium channel subunits Na_v_1.2 and leading to a gain-of-function (GoF), thereby causing increased neuronal excitability [14,15].

### 2.3. Benign (Familial) Infantile Epilepsy

BFIE occurs between 3 and 8 months of age, with clusters (8–10 per day) of repeated and brief episodes (2–5 min) over a few days [7]. Seizures remit spontaneously before the age of 3 years [16,17]. They are usually focal but can sometimes become generalized. Subjects present with motor arrest, unresponsiveness, head and/or eye deviation to one side, staring, fluttering of eyelids, grunting, cyanosis, diffuse hypertonia, and unilateral or bilateral clonic jerks of the limbs. Seizures are rarely observed later in life, although some subjects develop paroxysmal movement disorders such as paroxysmal dyskinesia, paroxysmal kinesigenic choreoathetosis, hemiplegic migraine, and episodic ataxia during adolescence [18].

BFIE is a genetically heterogeneous disease [19,20]. Most cases are associated with variants in the proline-rich transmembrane protein 2 (*PRRT2*) gene located at 16p11.2 [21,22]. *PRRT2* accounts for up to 70% of BFIE families [23,24]. This gene encodes a membrane protein that interacts with the presynaptic protein synaptosomal-associated protein 25 (SNAP-25), which is involved in releasing neurotransmitters from synaptic vesicles [25]. Variants have also been found in *SCN2A, SCN8A* (encoding the voltage-gated sodium channel subunits Na_v_1.6) [26], and on rare occasions in the *KCNQ2* and *KCNQ3* gene [20]. The overlapping phenotypes of these three syndromes make it difficult to distinguish between them, making genetic testing crucial.

### 2.4. GEFS+ Spectrum (Including Febrile Seizures Plus)

The most common seizure type in GEFS+ is classical febrile seizures, which occur after age 6 months and typically do not persist beyond 6 years of age. In comparison, febrile seizures plus (FS+), which is the second most common phenotype, occur prior to age 6 months and persist beyond 6 years of age [27]. Afebrile seizure types such as absence, myoclonic seizures, and atonic seizures may develop at various ages [27]. Prolonged and recurrent hemiclonic/focal clonic seizures provoked by fever and occurring prior to 6–9 months of life should raise a suspicion of Dravet syndrome.

GEFS+ is a genetically heterogeneous disorder. *SCN1A* (encoding the voltage-gated sodium channel subunits Na_v_1.1) accounts for the largest fraction, with disease-causing variants detected in approximately 10–20% of families [28,29]. Other genes such as *GABRA1, GABRB3, GABRG2, SCN1B,* and *STX1B* have also been linked to the syndrome [28,29,30,31,32]. Although inheritance is usually autosomal dominant with variable expressivity and penetrance, de novo pathogenic variants have also been described [28].

DEEs, epilepsy with myoclonic–atonic seizures, and Dravet syndrome belong to the severe end of the GEFS+ spectrum. Intra-familial phenotypical heterogeneity has been reported and on rare occasions, some affected members of a GEFS+ family may evolve into this severe end of the spectrum [33]. Factors underlying the mechanism of pleiotropy are not fully understood but may involve variable expressivity of a single disease-causing gene, genetic modifiers, or epigenetic factors.

### 2.5. Myoclonic Epilepsy in Infancy

Myoclonic seizures are the hallmark of this syndrome and involve the head and the upper limbs. They are typically present both in wakefulness and sleep and may occur several times daily. Seizures may be triggered by sudden noise, touch, or startle [34]. Onset tends to be between the ages of 4 months and 3 years, with a peak age of 6–18 months [34]. Development prior to seizure onset is typically normal. Febrile seizures can either precede or follow myoclonic seizures [34]. Seizures tend to remit within 6 months to 5 years from onset, but approximately 10% develop other epilepsies in late childhood or adolescence, mostly juvenile myoclonic epilepsy [34]. Although causal genes have yet to be identified, a family history of epilepsy or febrile seizures is reported in approximately 10% of subjects [34].

## 3. Developmental and Epileptic Encephalopathies (DEEs)

The term epileptic encephalopathy is used for conditions associated with normal development prior to the onset of epileptic activity but where the epileptic activity “per se” causes a progressive brain dysfunction [35,36]. In “developmental encephalopathies” (DE), an underlying genetic condition causes neurological impairment without the epileptic activity associated with regression or further slowing of development [35,36]. Developmental encephalopathies are often accompanied by epilepsies but typically as few and treatable seizures where the epileptic activity does not impair development or cognition [35]. In DEEs, both the underlying genetic condition and the epileptic activity are considered to play a role [35,36].

DEEs comprise a large, heterogeneous group of severe epilepsies characterized by early onset and treatment resistant epilepsy, multiple seizure types, frequent interictal epileptiform discharges on EEG, and developmental slowing or regression [37]. More than 100 genes including *SCN1A, SCN2A, SCN8A, KCNA2, KCNB1, KCNQ2, KCNT1, CACNA1A, STXBP1, CDKL5, PCDH19,* and *SLC2A1* have been associated with early-onset DEEs [37,38] (Table 1 and Table 2—lists not exhaustive). While some genes just have a single phenotype, others have a varying phenotype depending on the subject’s age and the nature of the causative variant(s). DEEs encompass several age-related electroclinical syndromes with specific seizure types and EEG features [37]. Ohtahara syndrome and early myoclonic encephalopathy are the earliest presenting of the epileptic encephalopathies and are also classified as early infantile DEEs. They are typically distinguished from each other according to specific clinical and etiologic criteria although considerable overlap exists [39].

Before describing the electroclinical syndromes that start during the first year of life, we must emphasize that most DEEs do not fit well within any of these electroclinical syndromes and can at best only be categorized as unclassified DEEs.

### 3.1. Ohtahara Syndrome

Ohtahara syndrome is characterized by onset of intractable seizures during the first few weeks to months of life [40]. Infants acutely develop focal or generalized tonic spasms that often occur in clusters. Spasms typically last up to 10 s and can occur hundreds of times per day [40,41]. Approximately one-third of subjects with Ohtahara syndrome will also develop other seizure types, most commonly focal onset motor seizures or generalized tonic-clonic seizures [40,42]. The EEG in Ohtahara syndrome indicates a burst suppression pattern, comprising bursts of high-amplitude spikes and polyspikes that alternate at a regular rate with periods of electric suppression [40]. The bursts coincide with the tonic spasms [43]. The prognosis is generally grave. Subjects with Ohtahara syndrome often die during infancy [42], and survivors invariably manifest a severe to profound psychomotor retardation [40,42]. Yamatogi and Ohtahara [42] showed that 75% of subjects later developed West syndrome, while 12% subsequently developed Lennox-Gastaut syndrome.

Structural brain anomalies, inborn errors of metabolism, and genetic abnormalities are amongst the causes of Ohtahara syndrome. To date, various genes that play essential roles in neuronal and interneuronal functions of the brain have been associated with Ohtahara syndrome [37,40]. Of these, syntaxin-binding protein 1 (*STXBP1*), which regulates the release of synaptic vesicles and the secretion of neurotransmitters, is the major contributor and explains ~30% of cases [38,44]. Pathogenic variants in other genes include the voltage-gated potassium channel gene (*KCNQ2)* (~20%) [38,45]; *SCN2A*, a gene encoding the voltage gated sodium channel Na_v_ 1.2 (~10%) [38,46] aristaless-related homeobox (*ARX*), a regulator of the proliferation and differentiation of neuronal progenitors [47]; solute carrier family 25 member 22 (*SLC25A22*), which encodes a mitochondrial glutamate transporter [48]; and potassium-activated channel subfamily T member 1 (*KCNT1)*, which is widely expressed in the nervous system [49,50] (See Table 1 for additional genes).

While protein-truncating variants in *KCNQ2* tend to cause benign familial epilepsy syndromes of infancy, missense variants may present with a distinct phenotype characterized by neonatal seizures that evolve to drug-resistant DEE and intellectual disability—*KCNQ2*-related neonatal epileptic encephalopathy (*KCNQ2*-NEE) (Table 2) [10,45]—although this entity does not always fulfill the clinical criteria for Ohtahara syndrome.

Age of seizure onset and seizure type are similar to BFNE, but the seizures are resistant to therapy. EEGs show a burst-suppression pattern during the first few days of life, followed by multifocal epileptiform activities [10]. During childhood, most subjects suffer from profound intellectual disabilities and neurological abnormalities such as hypotonia or spastic quadriplegia [10,51]. The wide spectrum of seizure types encompasses tonic (often focal) or apneic episodes, focal clonic activity, and autonomic changes. Similar to the *KCNQ2* variants, GoF *SCN2A* variants can also cause a spectrum of intractable childhood epilepsies ranging from Ohtahara syndrome and migrating focal seizures to epileptic spasms and Dravet syndrome [46]. The variants identified in intractable epilepsies alter the channel properties of Na_v_1.2 more than BFNIE variants do, suggesting the mechanism of more severe epileptic phenotypes [7,52].

### 3.2. Early Myoclonic Encephalopathy

Like Ohtahara syndrome, early myoclonic encephalopathy presents during the neonatal period—usually within the first 3 months of age, and sometimes as early as a few hours after birth. The initial presentation typically involves the onset of focal myoclonus, usually of the face or extremities. The jerks can shift from one area of the body to another in an asynchronous, seemingly random pattern. Tonic spasms are also frequent, occurring both isolated or in clusters [40]. The key EEG feature in early myoclonic encephalopathy comprises a suppression-burst pattern, much like that in Ohtahara syndrome [53]. However, in early myoclonic encephalopathy, the EEG pattern is either exclusively present or more distinct during sleep [53]. As in Ohtahara syndrome, the prognosis is poor with a high mortality and morbidity. Progressive, diffuse cortical atrophy is often present and suggestive of an underlying metabolic, degenerative, and/or genetic disorder [40,41,53]. The genes involved in this syndrome include the phosphatidylinositol glycan anchor biosynthesis class A (*PIGA)* involved in glycosylation [38,54,55], set-binding protein 1 (*SETBP1*) [38], salt-inducible kinase 1 (*SIK1*) [38,56], and *SLC25A22* [38,48] (Table 1).

### 3.3. Epileptic Spasms Syndrome and West Syndrome

West syndrome refers to the constellation of epileptic spasms (also known as infantile spasms), hypsarrhythmia, and developmental impairment, often with regression. In some infants, the developmental impairment may not be apparent or typical hypsarrhythmia is not present. This is often seen in subjects with early diagnosis and treatment [57]. This emphasizes the importance of early diagnosis, where a short lag time to therapy can lead to a better outcome [57].

The spasms usually begin in the first 4 to 6 months of life and are characterized by clusters of flexion or extension limb and trunk spasms. Developmental regression is often present. Spasms may be observed as the infant awakes from or falls to sleep and are accompanied by the EEG pattern of hypsarrhythmia [58]. The pattern consists of random, high-voltage, nonsynchronous spikes and slow waves of variable duration and topography [58]. The major causes include acquired brain injuries, congenital brain malformations, inborn error of metabolism, and pathogenic genetic variants [58]. Pathogenic variants have been detected in over 30 genes [7,38,59], and cyclin-dependent kinase-like 5 (*CDKL5*) (~10%), *STXBP1* (~2%), and *ARX* are among the most commonly detected genes (See Table 1 for additional genes). Although epileptic spasm syndrome is very heterogeneous, *TSC1* and *TSC2* (encoding tuberous sclerosis complex 1 and 2 subunits) cause a syndrome that is easily recognizable due to the associated brain MRI features. Approximately 30% of cases remain unexplained, suggesting that many causative genes have yet to be identified [59,60,61].

### 3.4. Dravet Syndrome (DS)

In DS or “severe myoclonic epilepsy of infancy”, healthy children typically develop seizures between the age of five to eight months [7,62,63]. Seizures may be triggered by fever, vaccination, stress, and bathing. Children experience prolonged hemiconvulsive or generalized febrile seizures during the first year of life [62,63]. This is followed by multiple types of intractable afebrile seizures, frequent occurrence of status epilepticus, and psychomotor retardation [63]. Interictal EEGs and brain imaging are usually normal at the time of onset, which often delays diagnosis. Although the vast majority of cases are caused by loss-of-function variants in *SCN1A* [64], other genes have also been reported to cause DS, including *SCN2A, SCN8A, SCN9A, SCN1B, PCDH19, GABRA1, GABRG2, STXBP1, HCN1, CHD2,* and *KCNA2* [64] (Table 1). Symptoms associated with *SCN1A* range from GEFS+ at the mild end to DS at the severe end [64].

### 3.5. Epilepsy of Infancy with Migrating Focal Seizures (EIMFS)

EIMFS is a rare DEE characterized by seizure migration between cerebral hemispheres and profound developmental impairment, often with regression [65]. Onset of seizures is typically during the first six months of life, and seizures are refractory to antiepileptic drugs. Typical interictal EEG features include multifocal spikes with slow background activity [66] while a burst-suppression pattern occurs on rare occasions [67]. EIMFS is currently associated with pathogenic variants in 33 genes [65]. Both autosomal dominant and recessive patterns of inheritance have been described [65]. The most commonly involved genes are *KCNT1* (in ~27%) and *SCN2A* (in ~7%) followed by *SCN1A* [65,68] (See Table 1 for additional genes). The genetic heterogeneity in EIMFS resembles that in other epileptic syndromes, such as epileptic spasms, but a considerably higher yield with current testing strategies has been described [65].

## 4. Advantages and Pitfalls in Genetic Testing of Subjects with Onset of Epilepsy Syndromes during the First Year of Life

**Figure 1:**
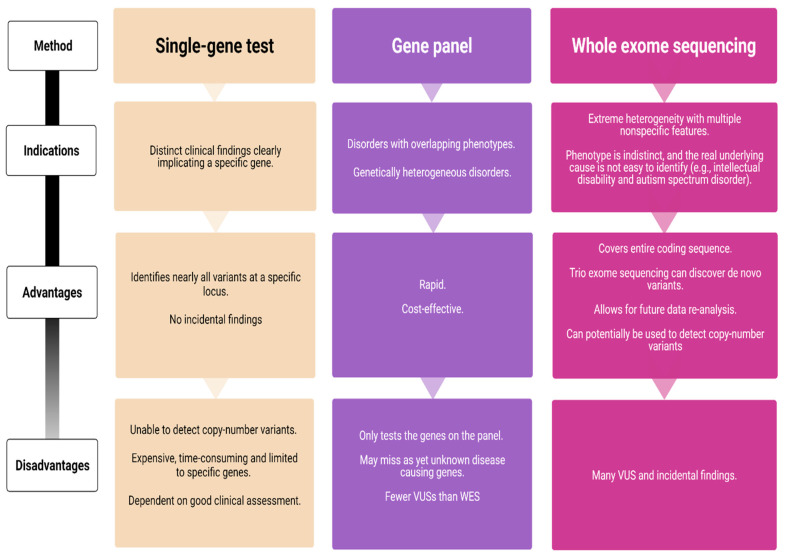
Comparison of genetic testing strategies (VUS = variant of unknown significance; WES = whole exome sequencing).

Over the last decade, next-generation sequencing (NGS) has led to remarkable advances in the field of epilepsy genetics. It has become a powerful tool for genetic testing and provides the foundation for optimized genetic counseling. NGS is largely available in many countries as part of the routine diagnostic workup, resulting in higher diagnostic yields and greater insights into the underlying disease mechanisms. Several NGS technologies are currently available—targeted gene panels, whole-exome sequencing (WES), and whole-genome sequencing (WGS) (Figure 1).

Single-gene sequencing is indicated in subjects with a likely well-characterized monogenic disorder and should be avoided if the disorder demonstrates a high degree of genetic heterogeneity. It is the gold standard for detecting small sequence variations, but as only one gene is screened at a time, Sanger sequencing can become a time-consuming and expensive odyssey. In contrast, gene panels enable simultaneous testing of multiple genes associated with heterogeneous infantile epilepsy syndromes. Recent studies have shown an overall diagnostic yield of epilepsy gene panels ranging 15–48% [4,69,70,71], but the results vary according to phenotype. The highest yield is among subjects with neonatal-onset epilepsies and epileptic encephalopathies (57%) [4]. Lindy et al. [69] evaluated the diagnostic outcomes from testing 70 genes in more than 8500 subjects with epilepsy and neurodevelopmental disorders. They used a gene panel combined with exon-level array comparative genomic hybridization (CGH) and found pathogenic or likely pathogenic variants in 15.4% of the subjects [69]. Although this was relatively low compared to previous studies, the results provide a robust estimate given the large subject cohort. In comparison, the diagnostic yield of WES is between 25% and 44% [72,73,74]. One disadvantage with gene panels is that they are restricted to the number of genes on a given panel and that disease-causing variants in unknown genes are missed, while WES covers the entire human coding sequence.

Between 5 and 10% of DEEs harbor a causative or potentially contributing copy number variant (CNV) and chromosomal microarrays studies are considered a standard early investigation [38,75] especially in exome negative subjects. CNVs may involve multiple genes and/or regulatory regions. Microdeletions and duplications can also represent as normal variants in the human karyotype, and pathogenecity must be ascertained once a CNV is detected. For this aim, several factors are taken into consideration: Segregation (as a CNV inherited from a healthy parent is less likely to be disease causing); presence of the CNV in the general population using gnomAD [76] or in affected subjects using gene variant databases such as ClinVar [77]; based on the function of genes contained by the CNV; and finally the size of the CNV (as deletions larger than 1 Mb are more likely to be causative) [38].

Although WES is not optimal for detecting CNVs, it has the potential to discover new genes—particularly when subjects have similar phenotypes that carry similar variants. A single WES or a larger gene panel increases the detection rate of variants of unknown significance (VUS), thus making parental testing, family-segregation analysis, and functional studies crucial. WES and WGS also pose a risk of detecting incidental but medically important findings that are unrelated to the indication for ordering the test. Targeted NGS requires confirmation using a second method, and complimentary Sanger sequencing is often required due to the low coverage of exons.

A clear advantage of WES/WGS is the possibility to re-analyze the data. As new epilepsy genes are constantly being discovered, many laboratories offer re-analysis after certain time intervals. A WES initially considered as uncertain or negative may yield a diagnosis after a period of 6–12 months [78].

Genetic testing as part of a routine diagnostic workup is also useful in adults or elderly subjects diagnosed with early-onset epilepsy [79,80]. Investigation of such cohorts have taught us that, even in adulthood, a precision diagnosis can lead to optimized therapeutic decision-making, improved seizure control, and potentially a better quality of life [80].

## 5. Utility of Early Genetic Testing for Precision Therapy Approaches

**Figure 2:**
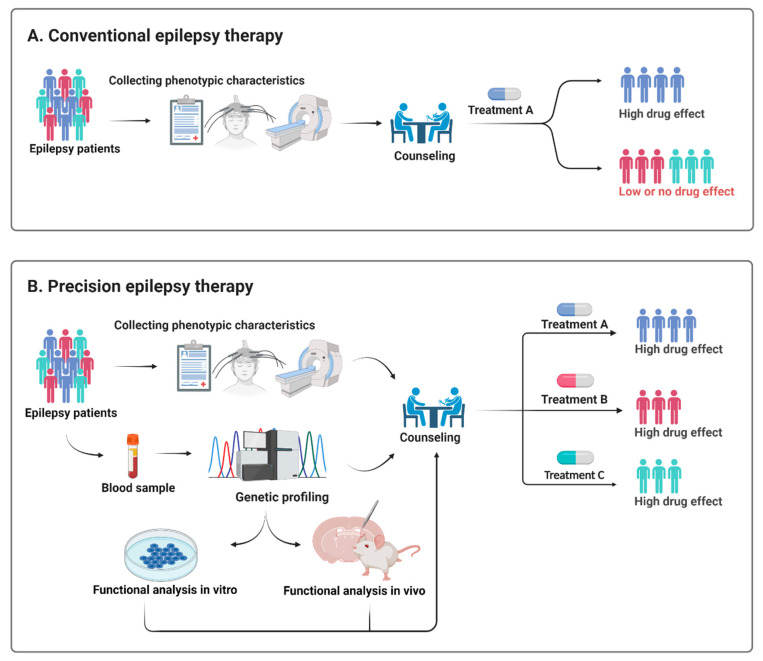
Schematic illustration of how a precision therapy could be applied in epilepsy. Treatment of epilepsy remains largely empirical and rather than the present trial and error approach (**A**), genetic testing and preclinical in vitro and in vivo models may enable healthcare providers to select a more targeted treatment approach (**B**).

Treatment of epilepsy remains largely empirical and is often a trial and error rather than a targeted treatment approach (Figure 2). Early genetic testing lays the foundation for a precision diagnosis and thereby precision therapy, but it needs to be backed up by functional testing to provide pathogenicity and to explore the underlying functional effect of a given variant [81] (Figure 2).

Ion channel disorders, in particular, have taught us how crucial it is to have insight into the loss-of-function (LoF) versus GoF pathomechanisms when forming therapeutic decisions.

*SCN1A* is a commonly detected epilepsy gene that has an estimated incidence of 1 in 12,200 live births [82]. The most frequent *SCN1A*-related disorder is DS, and *SCN1A*-deficiency is also by far the most common genetic cause of DS [64]. In *SCN1A-*related DS, sodium channel blockers such as lamotrigine and carbamazepine should be avoided as they may aggravate seizures, while fenfluramine, cannabidiol, valproic acid, topiramate, clobazam, and stiripentol can be beneficial [62,83].

Another example of precision therapy would be *SCN2A*-deficiency. GoF *SCN2A* variants present with seizures within the first 3 months of life—Ohtahara syndrome, EIMFS, and unclassified DEEs at the severe end of the spectrum and BFNIE at the mild end [46]. In contrast, LoF variants either cause non-epilepsy phenotypes such as non-syndromic intellectual disability, autism, and episodic ataxia or late-onset phenotypes (>3 months of life) including West syndrome, myoclonic atonic epilepsy, and unclassified DEEs [46]. Despite the lack of personalized treatment options in LoF *SCN2A* cases (other than avoiding sodium channel blockers), GoF variants in *SCN2A* might respond to sodium channel blockers [83,84]. Other examples include ketogenic diet in *SLC2A1*-deficiency [83], retigabine in LoF *KCNQ2-* and *KCNQ3-*related disorders [85], carbamazepine in *PRRT2*-deficiency [83], quinidine in *KCNT1*-encephalopathies, and ganaxolone in *CDKL5-* and *PCDH19-*related disorders [83,86].

A recent study showed that the seizure type at presentation in neonates could suggest a genetic etiology [87]. This was achieved by comparing 20 neonates with a verified genetic epilepsy to 40 neonates with acute provoked seizures [87]. The genetic epilepsies were primarily caused by pathogenic variants in genes encoding ion channels [87]. While neonates with acute provoked seizures often presented with clonic seizures, tonic seizures were mainly associated with channelopathies and were often controlled by sodium channel-blockers [87]. This study suggests that early identification of the seizure type can prompt appropriate workup and treatment [87]. It potentially guides treatment while waiting for results of genetic testing, but it also provides a potentially useful tool in countries where the access to genetic testing is currently limited.

At present, disease-specific treatments are only available for a minority of the severe genetic epilepsies and the majority of these are antiseizure medications. Future cutting-edge therapies should target not only seizures but also the developmental outcome and comorbidities [38]. Promising therapies include antisense oligonucleotide (ASO) modulation and, ultimately, gene therapy acting directly on the underlying mechanism that causes the widespread effects of the disorder, as they are likely to be more extensive than those attributed to the epilepsy alone [38]. Two such examples are ASOs used in mice models of *SCN1A-* and *SCN8A-*encephalopathy and DS [88,89]. An open-label phase 1b/2a trial with an ASO increasing functional *SCN1A* mRNA and protein expression has recently started and is including subjects with DS second to pathogenic *SCN1A* variants (https://clinicaltrials.gov/ct2/show/NCT04442295, accessed on 22 June 2020 and 4 May 2021). Future clinical trials for genetic therapies must be designed as neurodevelopmental disorder trials rather than epilepsy trials and should reliably measure non-seizure outcomes.

Thus, greater knowledge about genetics and biochemistry is needed amongst healthcare providers before precision medicine approaches can become a part of routine healthcare. In the future, physicians and other healthcare providers will increasingly find themselves needing to interpret the results of genetic tests, understand how that information is relevant to treatment or prevention approaches, and convey this knowledge to subjects.

## 6. Genetic Testing in Self-Limiting Epilepsies

One could argue why early genetic testing and precision diagnosis is needed for self-limiting epilepsies. It is not difficult to imagine the massive toll on parents when their newborns/infants experience convulsions and are hospitalized even in familial cases. Early genetic testing leading to precision diagnosis and an optimal genetic counseling empowers both caregivers and healthcare providers. It provides an explanation and certainty; it enables a more targeted genetic counseling, including knowledge about the prognosis and recurrence risk. This is particularly useful for those with neonatal seizures caused by *KCNQ2-* or *KCNQ3-*deficiency as post-infantile seizures occur in up to 30% of cases [13]. Furthermore, precision diagnosis allows the subject and families to enter gene-specific networks of families with the same genetic condition. Importantly, early genetic testing enables subjects and caregivers to receive tailored treatment and prevent unnecessary and potentially harmful diagnostic procedures and managements. One such example is carbamazepine, which is considered as a precision therapy in *PRRT2*-deficiency [83].

## 7. Conclusions

Epilepsy syndromes that start in the first year of life display phenotypic and genetic heterogeneity, and genetic testing is crucial for subjects, caregivers, and clinicians. The availability and reduced cost of genetic testing will enable us to make early and accurate diagnosis in pediatric epilepsies, which will inevitably lead to optimized and tailored treatment approaches. Due to the clinical heterogeneity of gene panels targeted at monogenic epilepsies, WES and WGS should be the preferred method compared to a single-gene approach that has limited usefulness in epilepsy genetics. Clinicians will need to consider both the advantages and the pitfalls of the available genetic tests before choosing the path to follow. Early genetic testing enables precision diagnosis and may ultimately lead to a disease-specific treatment.

## Figures and Tables

**Table 1:** Epilepsy syndromes with onset during the neonatal period and/or infancy and the commonly associated genes (list is not exhaustive). The most commonly occurring genes within each electroclinical syndromes are listed first with percentages in bracket followed by less commonly occurring genes listed in alphabetic order and according to inheritance.

Epilepsy Syndrome		Gene Involved	Protein Product	Mode of Inheritance
Benign familial epilepsy syndromes	BFNE	*KCNQ2*	Subunit of voltage-gated K+ channel	AD
		*KCNQ3*	Subunit of voltage-gated K+ channel	AD
	BFNIE	*SCN2A*	Subunit of voltage-gated Na+ channel	AD
	BFIE	*PRRT2*	Protein-rich transmembrane protein 2	AD
		*SCN2A*	Subunit of voltage-gated Na+ channel	AD
		*SCN8A*	Subunit of voltage-gated Na+ channel	AD
	GEFS+	*SCN1A*	Subunit of voltage-gated Na+ channel	AD
		*SCN1B*	Subunit of voltage-gated Na+ channel	AD, AR
		*GABRG2*	Subunit of GABAa receptor	AD
		*STX1B*	Syntaxin 1B	AD
	Myoclonic epilepsy in infancy	Unknown	-	-
Developmental and epileptic encephalopathies	Ohtahara syndrome	*STXBP1* (in ~30%)	Syntaxin binding protein 1	AD
		*KCNQ2* (in ~20%)	Subunit of voltage-gated K+ channel	AD
		*SCN2A* (in ~10%)	Subunit of voltage-gated Na+ channel	AD
		*GNAO1*	Guanine nucleotide-binding protein, α-activating activity polypeptide O	AD
		*KCNT1*	Subunit of voltage-gated K+ channel	AD
		*SNC8A*	Subunit of voltage-gated NA+ channel	AD
		*SIK1*	Salt-inducable kinase 1	AD
		*AARS*	Alanyl-tRNA synthetase 1	AR
		*BRAT1*	BRACT1-associated atm activator 1	AR
		*CACNA2D2*	Calcium channel, voltage-dependent, α-2/delta subunit 2	AR
		*NECAP1*	NECAP endocytosis-associated protein 1	AR
		*PIGQ*	Phosphatidylinositol glycan anchor biosynthesis class Q protein	AR
		*SLC25A22*	Solute carrier family 25, member 22	AR
		*ARX*	Aristaless-related homeobox	XR
		*PIGA*	Phosphatidylinositol glycan anchor biosynthesis class A protein	XR
	Early myoclonic encephalopathy	*SETBP1*	SET-binding protein 1	AD
		*SIK1*	Salt-inducable kinase 1	AD
		*SLC25A22*	Solute carrier family 25, member 22	AR
		*PIGA*	Phosphatidylinositol glycan anchor biosynthesis class A protein	XR
	Infantile spasms syndrome (including WEST syndrome)	*STXBP1* (in ~2%)	Syntaxin binding protein 1	AD
		*CHD2*	Chromodomain helicase DNA-binding protein 2	AD
		*DNM1*	Dynamin 1	AD
		*FOXG1* (duplications)	Forkhead box G1	AD
		*GABRA1*	Subunit of GABAa receptor	AD
		*GABRB3*	Subunit of GABAa receptor	AD
		*GABRG2*	Subunit of GABAa receptor	AD
		*GNAO1*	Guanine nucleotide-binding protein, α-activating activity polypeptide O	AD
		*GRIN1*	Glutamate receptor, ionotropic, n-methyl-d-aspartate, subunit 1	AD, AR
		*GRIN2A*,	Glutamate receptor, ionotropic, n-methyl-d-aspartate, subunit 2A	AD
		*GRIN2B*	Glutamate receptor, ionotropic, n-methyl-d-aspartate, subunit 2B	AD
		*HCN1*	Hyperpolarization activated cyclic nucleotide gated potassium channel 1	AD
		*KCNA2*	Subunit of voltage-gated K+ channel	AD
		*KCNT1*	Subunit of voltage-gated K+ channel	AD
		*MAGI2*	Membrane-associated guanylate kinase, WW and PDZ domains-containing 2	AD
		*MEF2C*	MADS BOX transcription enhancer factor 2, polypeptide C	AD
		*NDP*	Norrin cystine knot growth factor NDP	AD
		*PTEN*	Phosphatase and tensin homolog	AD
		*SCA2*	Spinocerebellar ataxia 2	AD
		*SPTAN1*	Spectrin, α, non-erythrocytic 1	AD
		*SETBP1*	SET-binding protein 1	AD
		*SIK1*	Salt-inducable kinase 1	AD
		*SCN1A*	Subunit of voltage-gated Na+ channel	AD
		*SCN1B*	Subunit of voltage-gated Na+ channel	AD, AR
		*SCN2A*	Subunit of voltage-gated Na+ channel	AD
		*SCN8A*	Subunit of voltage-gated Na+ channel	AD
		*SCN9A*	Subunit of voltage-gated Na+ channel	AD
		*STXBP1*	Syntaxin binding protein 1	AD
		*TCF4*	Transcription factor 4	AD
		*TSC1*	Tuberous sclerosis complex 1	AD
		*TSC2*	Tuberous sclerosis complex 2	AD
		*DOCK7*	Dedicator of cytokinesis 7	AR
		*NRXN1*	Neurexin 1	AR
		*PIGN*	Phosphatidylinositol glycan anchor biosynthesis class N protein	AR
		*PIGP*	Phosphatidylinositol glycan anchor biosynthesis class P protein	AR
		*PIGQ*	Phosphatidylinositol glycan anchor biosynthesis class Q protein	AR
		*PIGS*	Phosphatidylinositol glycan anchor biosynthesis class S protein	AR
		*PLCB1*	Phospholipase C, β-1	AR
		*SLC25A22*	Solute carrier family 25, member 22	AR
		*ST3GAL3*	ST3 β-galactoside α-2,3-sialyltransferase 3	AR
		*TBC1D24*	TBC1 domain family, member 24	AR
		*WWOX*	WW domain-containing oxidoreductase	AR
		*CDKL5*	Cyclin-dependent kinase-like 5	XD
		*ARX*	Aristaless-related homeobox	XR
		*PIGA*	Phosphatidylinositol glycan anchor biosynthesis class A protein	XR
		*ALG13*	ALG13 UDP-N-acetylglucosaminyltransferase subunit	XL
		*PCDH19*	Protocadherin subclass of the cadherin superfamily	XL
	Dravet/dravet-like phenotypes	*SCN1A* (in ~90%)	Subunit of voltage-gated Na+ channel	AD
		*CHD2*	Chromodomain helicase DNA-binding protein 2	AD
		HCN1 *	Hyperpolarization activated cyclic nucleotide gated potassium channel 1	AD
		*GABRA1*	Subunit of GABAa receptor	AD
		*GABRB3*	Subunit of GABAa receptor	AD
		*GABRG2*	Subunit of GABAa receptor	AD
		*KCNA2*	Subunit of voltage-gated K+ channel	AD
		*SCN1B*	Subunit of voltage-gated Na+ channel	AD, AR
		*SCN2A*	Subunit of voltage-gated Na+ channel	AD
		*SCN8A*	Subunit of voltage-gated Na+ channel	AD
		*SCN9A*	Subunit of voltage-gated Na+ channel	AD
		*STXBP1*	Syntaxin binding protein 1	AD
		PCDH19 *	Protocadherin subclass of the cadherin superfamily	XL
	EIMFS	*KCNT1* (in ~27%)	Subunit of voltage-gated K+ channel	AD
		*SCN2A* (in ~7%)	Subunit of voltage-gated Na+ channel	AD
		*SCN1A*	Subunit of voltage-gated Na+ channel	AD
		*GABRA1*	Subunit of GABAa receptor	AD
		*GABRB1*	Subunit of GABAa receptor	AD
		*GABRB3*	Subunit of GABAa receptor	AD
		*HCN1*	Hyperpolarization-activated cyclic nucleotide-gated potassium channel 1	AD
		*KCNQ2*	Subunit of voltage-gated K+ channel	AD
		*SCN8A*	Subunit of voltage-gated Na+ channel	AD
		*ATP1A3*	ATPase, Na+/K+ transporting, α-3 polypeptide	AD
		*AIMP1*	Aminoacyl-tRNA synthetase complex-interacting multifunctional protein 1	AR
		*BRAT1*	BRCA1-associated ATM activator 1	AR
		*ITPA*	Inosine triphosphatase	AR
		*KARS*	Lysyl-tRNA synthetase 1	AR
		*PLCB1*	Phospholipase C, β-1	AR
		*QARS*	Glutaminyl-tRNA synthetase 1	AR
		*SLC12A5*	Solute carrier family 12	AR
		*SLC25A22*	Solute carrier family 25, member 22	AR
		*TBC1D24*	TBC1 domain family, member 24	AR
		*WWOX*	WW domain-containing oxidoreductase	AR
		*CDKL5*	Cyclin-dependent kinase-like 5	XD
		*SMC1A*	Structural maintenance of chromosomes 1A	XD
		*PIGA*	Phosphatidylinositol glycan anchor biosynthesis class A protein	XR

Abbreviations: AD = autosomal dominant; AR = autosomal recessive; BFIE = benign familial infantile epilepsy; BFNE = benign familial neonatal epilepsy; BFNIE = benign familial neonatal infantile epilepsy; BRCT1 = breast cancer associated ATM activator 1; EIMFS = early infantile migrating focal seizures; GABA = γ aminobutyric acid; GEFS+ = genetic epilepsy with febrile seizures plus; XD = X-linked dominant; XL = X-linked; XR = X-linked recessive. * In most subjects, the syndrome is distinguishable from Dravet syndrome.

**Table 2:** Genotype–phenotype relationship of the most recurrent genes associated with benign familial neonatal and/or infantile epilepsies and early-onset developmental and epileptic encephalopathies together with potential precision therapy approaches.

Gene	Associated Epilepsy Syndromes	Associated with Structural Brain Anomalies/Lesions	Potential Therapeutic Approaches
*ARX*	Ohtahara syndrome	Yes	Currently none available
	Epileptic spasms syndrome		
	Myoclonic epilepsy		
	Nonsyndromic intellectual disability with or without epilepsy		
	Developmental and epileptic encephalopathy 1		
*CDKL5*	Epileptic spasms syndrome	Yes	Ganaxolone (PMID 33165915)
	Developmental and epileptic encephalopathy 2		
*KCNA2*	Developmental and epileptic encephalopathy 32	Yes	GoF: 4-Aminopyridine Sodium channel blockers (PMID 33515866)
	ESES		
*KCNB1*	Developmental and epileptic encephalopathy 26	Yes	Currently none available
	Epileptic spasms syndrome		
	ESES		
*KCNQ2*	Benign familial neonatal epilepsy	Yes	LoF: Sodium channel blockers (PMID 24371303 and 25880994) Retigabine (PMID 27602407)
	Ohtahara syndrome		
	Neonatal epileptic encephalopathy		
	EIMFS		
	Developmental and epileptic encephalopathy 7		
*KCNQ3*	Benign familial neonatal epilepsy	Yes	LoF: Sodium channel blockers (PMID 27888506) Retigabine (PMID 27602407)
*KCNT1*	Autosomal dominant sleep-related hypermotor epilepsy	Yes	GoF: Quinidine (PMID 31054119)
	EIMFS		
	Developmental and epileptic encephalopathy 14		
*PRRT2*	Benign familial infantile epilepsy	Yes	Carbamazepine (PMID 32413583)
	Infantile convulsion and choreoathetosis syndrome		
	Paroxysmal kinesigenic dyskinesia		
*SCN1A*	Dravet syndrome	Yes	Lof: Stiripentol (+ valproate + clobazam) Fenfluramine Cannabidiol Avoid sodium channel blockers (PMID 32413583)
	Genetic epilepsy with febrile seizure plus		
	EIMFS		
	Developmental and epileptic encephalopathy 6		
*SCN2A*	Benign familial neonatal infantile epilepsy	Yes	GoF: Sodium channel blockers (PMID 32413583) LoF: Avoid sodium channel blockers (PMID 32413583)
	Genetic epilepsy with febrile seizures plus		
	Epileptic spasms syndrome		
	EIMFS		
	Ohtahara syndrome		
	Developmental and epileptic encephalopathy 11		
*SCN8A*	Benign familial neonatal infantile epilepsy	Yes	GoF: Sodium channel blockers (PMID 32413583) LoF: Avoid sodium channel blockers (PMID 32413583)
	Developmental and epileptic encephalopathy 13		
*STXBP1*	Ohtahara syndrome.	Yes	Levetiracetam may have superior effect on seizures and movement disorder (PMID 29896790).
	Epileptic spasms syndrome		
	Nonsyndromic intellectual disability with or without epilepsy		
	Developmental and epileptic encephalopathy 4		
*SLC25A22*	Ohtahara syndrome	Yes	Currently none available
	Early myoclonic epilepsy		
	EIMFS		
	Developmental and epileptic encephalopathy 3		
*TSC1*	Epileptic spasms syndrome	Yes	Everolimus and other mTOR inhibitors (PMID 27351628)
*TSC2*	Epileptic spasms syndrome	Yes	Everolimus and other mTOR inhibitors (PMID 27351628)

Abbreviations: EIEE = early infantile epileptic encephalopathy; EIMFS = early infantile focal migrating seizure; ESES = electroclinical status epilepticus in sleep; GoF = gain-of-function; LoF = loss-of-function.

## Data Availability

Not applicable.
